# The Nuchal Cord Conundrum: Understanding and Addressing Umbilical Entanglement in the Third Trimester of Pregnancy

**DOI:** 10.3390/jcm13226836

**Published:** 2024-11-14

**Authors:** Julia Murlewska, Sławomir Witkowski, Maria Respondek-Liberska, Iwona Strzelecka

**Affiliations:** 1 Department of Prenatal Cardiology, Polish Mother’s Memorial Hospital Research Institute, 93-338 Lodz, Polandi.j.strzelecka@gmail.com (I.S.); 2 Medical Faculty, Ludwik Rydygier Collegium Medicum Bydgoszcz, 85-067 Bydgoszcz, Poland; 3 Department of Diagnoses and Prevention of Fetal Malformations of Medical, University of Lodz, 90-136 Lodz, Poland

**Keywords:** nuchal cord, umbilical cord, Tei index, prenatal echocardiography, vagus nerve stimulation

## Abstract

**Background/Objectives:** Better understanding of and addressing umbilical entanglement in the third trimester of pregnancy is necessary to estimate its impact on fetal circulation. An analysis of single physiological pregnancies wrapped with one or two coils of the umbilical cord around the neck makes it possible to understand the severity of the problem and distinguish it from perinatal umbilical strangulation. **Methods:** In an echocardiographic study performed at 32.1 weeks of pregnancy in fetuses with one and two coils of the umbilical cord around the neck, the pulsatility index (PI) and the Tei index for the left (Tei LV) and right ventricle (Tei RV) of the heart were measured to evaluate cardiac function. **Results:** The study showed significantly higher Tei RV and Tei LV for fetuses with one (93 cases) and two coils of the umbilical cord around the fetal neck (26 cases) with respect to the control group of fetuses (680 cases) with no umbilical cord around the fetal neck, whereas PI UMBA did not differ significantly. **Conclusions:** Wrapping of the umbilical cord around the fetal neck may affect the study of the fetal heart without any mechanically induced compression of the umbilical vessels in normal pregnancy.

## 1. Introduction

Wrapping of the fetal neck with the umbilical cord has not been of particular interest to scientists so far. No European or American recommendations [1,2,3,4] point out the need for ultrasound diagnostics of the umbilical cord in terms of its entanglement around the fetal neck. This problem is rather considered in the context of childbirth and postpartum complications [5]. Single cases of cord compression around the neck are described and refer to numerous hemorrhagic lesions: bleeding, edema of the scrotum, neck ecchymosis in the epigastric area over the umbilical ring, and extravasation to the white of the eye with small petechiae on the skin of the face [6]. In addition, umbilical cord compression is of great interest to skilled attorneys who engage in the analysis of the course of pregnancy and childbirth in terms of medical malpractice [5,6,7,8,9]. In addition to the interest in umbilical strangulation in newborns during childbirth, the literature is full of interesting presentations of the cases of fetuses grabbing and sucking the umbilical cord or reacting with hiccups when wrapped with the umbilical cord. However, all this is presented in the context of cardiotocographic rather than echocardiographic monitoring [10,11,12,13,14]. Wharton’s jelly is a gelatinous substance that surrounds the umbilical vessels (umbilical arteries and vein) in the umbilical cord. Wharton’s jelly is a crucial component of the umbilical cord, providing protection for the umbilical vessels against potential mechanical compression. This gel-like substance is rich in hyaluronic acid and chondroitin sulfate, contributing to its unique viscosity and elasticity. It plays a vital role during pregnancy and supplies blood to the developing fetus even if the fetus moves within the amniotic cavity [15]. Our study attempts to answer the question of how wrapping of the fetus with the umbilical cord around the neck affects its peripheral and intracardiac blood flows assessed by the flow resistance parameter in the umbilical blood and the systolic and diastolic Tei index of the fetal heart.

## 2. Materials and Methods

**Figure 1:**
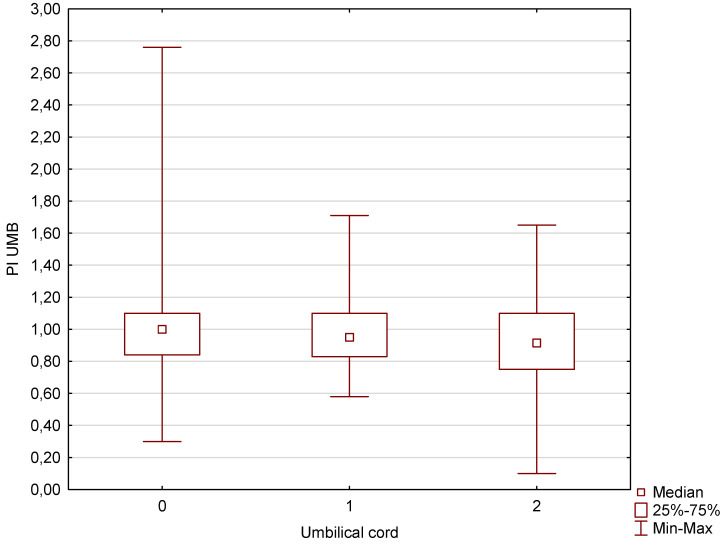
A box graph of PI UMBA in a group of fetuses without and with one or two coils of the umbilical cord around the fetal neck.

This was an analysis of a group of fetuses subject to ultrasound examinations at the tertiary center in the period between 2018 and 2023. All the information required for this study was retrieved from the database of our unit. The study involved single pregnancies. Healthy fetuses were considered fetuses with normal biometry and normal heart anatomy without extracardiac malformations. A total number of 3791 patients were analyzed for the purposes of this study. However, 2988 fetuses were excluded because their gestational age was under 27 weeks. Fetuses with three umbilical coils around the neck were also excluded due to the fact that there were only 3 such cases. The fetuses with single umbilical artery (SUA) (304 SUA cases without nuchal cord and 1 case with nuchal cord) were excluded as well. All the measurements of the pulsatility index (PI) in the umbilical cord and Tei index for left and right ventricles were performed by experienced prenatal echocardiographers. The selected 803 fetuses were distributed into four groups. In group A (control group), there were 680 fetuses without the umbilical cord around the fetal neck. In group B (study group B), there were 93 fetuses with one coil of the umbilical cord around the fetal neck. In group C (study group C), there were 26 fetuses with two coils of the umbilical cord around the fetal neck. The gestational age of the patients ranged from 27 weeks to 40 weeks according to the ultrasound biometry, which was based on the measurement of the abdominal circumference, head circumference, and femur length, and there were no statistical differences between the analyzed groups A, B, C (*p* > 0.05). An umbilical nuchal cord forming a U shape was diagnosed with color Doppler on a longitudinal and transverse scan of the fetal neck. A U-shaped nuchal cord refers to a situation where the umbilical cord is wrapped around the fetal neck in a U-shaped configuration. This means that the umbilical cord forms a loop around the neck of the fetus, which resembles the letter “U” [16,17]. Attached Supplementary clips S1–S3 show how the U shape of the umbilical cord around the neck of the fetus should be detected by Doppler imaging. Statistical analysis was performed using TIBCO Statistica 13.3 and Microsoft Excel 365 software. Box graphs of the Tei index for the right and left ventricles were created for groups A, B, C. The data were not normally distributed (Shapiro–Wilk test *p* < 0.05). The Kruskal–Wallis test was used for preparing the statistical analysis of the analyzed groups of fetuses. A *p*-value of < 0.05 was considered statistically significant. In our study, we focused on the pulsatility index for the umbilical artery (PI UMBA) (Figure 1) and the Tei index for the left and right ventricles of the heart. These parameters are crucial for assessing fetal well-being and cardiac function, and we believe our findings will contribute significantly to this field. We acknowledge that there are other important parameters such as the pulmonary trunk width, aorta size, fetal weight, amniotic fluid index, and pulsatility index for the middle cerebral artery (PI MCA), which we have detailed in our previous paper in Diagnostics in 2023 [18]. However, we have chosen not to discuss these in this study to maintain a focused scope on the PI UMBA and Tei index.

## 3. Results

**Figure 2:**
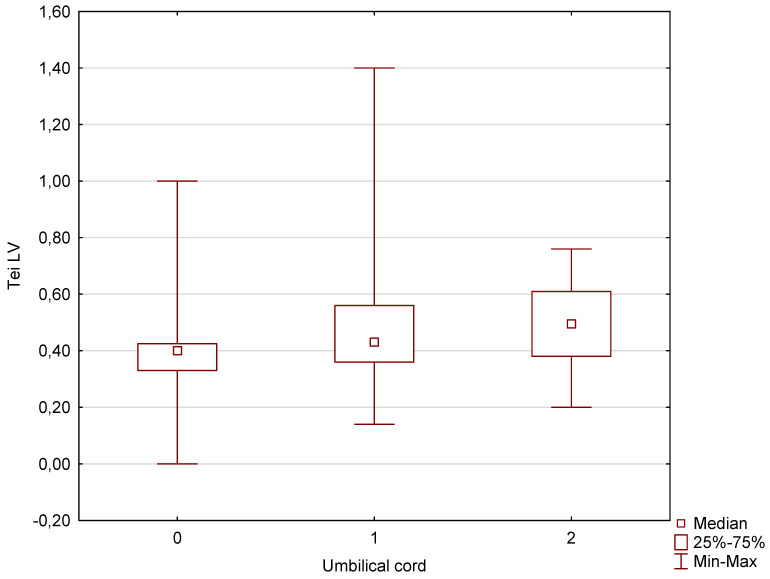
A box graph of the Tei index for the left ventricle in a group of fetuses without and with one or two coils of the umbilical cord around the fetal neck.

**Figure 3:**
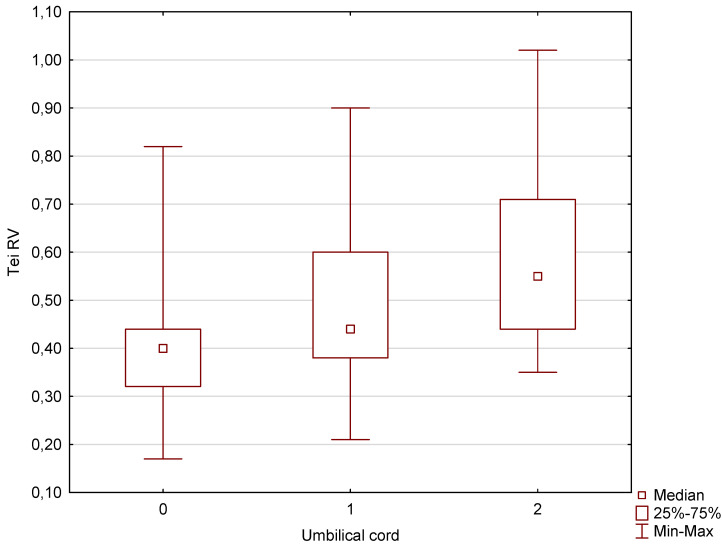
A box graph of the Tei index for the right ventricle in a group of fetuses without and with one or two coils of the umbilical cord around the fetal neck.

Our analysis of the pulsatility index, which constitutes an umbilical artery Doppler parameter that assesses the degree of resistance in the umbilical cord, showed that in the case of fetuses wrapped once or twice with the umbilical cord in the third trimester of pregnancy with an average gestational age of 33 weeks and 1 day, no significantly higher parameters were obtained for those fetuses with no mechanical compression of the umbilical cord. The opposite dependencies, on the other hand, were obtained in the Tei index parameters that assess the systolic and diastolic function of the heart chambers. In this case, these parameters were significantly higher not only in fetuses with two coils of the umbilical cord around the fetal neck, but also with one coil. These results are presented in detail below in graphical and tabular forms (Figure 2 and Figure 3, Table 1 and Table 2). The explanation of these differences can be found in the discussion section. 

## 4. Discussion

Our results have confirmed that wrapping of the fetus with the umbilical cord around the neck does not always involve mechanical compression (Figure 3, Table 2). This is due to the fact that the umbilical cord in a physiological pregnancy is protected by Wharton’s jelly. We obtained the same results in our previous study [19], where the PI values for the umbilical artery (PI UMBA) were 1.15 ± 0.31 vs. 1.09 ± 0.25 for fetuses wrapped with one coil of the umbilical cord around the neck compared to the control group of fetuses from pregnancies with a normal physiological course without associated defects, structural abnormalities, or growth disorders or chronic maternal diseases such as hypertension or diabetes. The differences between the PI UMBA values were not statistically significant in our previous study, which involved a total number of 46 fetuses wrapped around the neck and 70 fetuses from the control group. The same results have been obtained in our other study on a larger group of patients [19]. The elaboration published in Diagnostics in 2023 [18] included an even larger group of patients—854 fetuses from 33-week pregnancies—and also did not show the occurrence of any features of peripheral gradual compression of the umbilical vessels. According to the article [18], the PI UMBA values were, respectively, 0.974 ± 0.2 vs. 1.101 ± 0.2 vs. 0.889 ± 0.3 for fetuses wrapped with two coils of the umbilical cord versus fetuses wrapped with one coil of the umbilical cord compared with fetuses from the control group [18]. Although in the previous studies, LGA (large for gestational age) and SGA (small for gestational age) fetuses were analyzed [18], any significant additional changes in FHR (fetal heart rate) and AFI index were not observed, and the obstetric results of these cases were normal. There were no cases of newborn respiratory acidosis and newborn hypoxia as well as no prematurity or low birth weight; however, a higher percentage of cesarean sections (54.5% vs. 31.4%; *p* = 0.014) and persistent umbilical cord strangulation during childbirth (37% vs. 18.6%; *p* = 0.027) were observed [18]. These results of our other publications [18,19] as well as this study convince us that in the absence of growth disorders and oligohydramnios, fetuses wrapped with an umbilical cord around the neck, even several times, should not be subject to worsening of umbilical artery Doppler parameters, which is rather the case of higher lean umbilical cords with decreased umbilical vein caliber in intrauterine growth-restricted fetuses [20]. Potentially significantly higher PI UMBA values may be obsered in SGA fetuses who are wrapped with the umbilical cord around the neck [19,21] and although we had too few such cases to assess this by statistical methods, it can be said that practically every SGA case studied had the umbilical cord wrapped around the neck [21].

Another issue of our elaboration raised in the discussion should be related to the Tei index parameters, which were studied in order to assess the impact of umbilical cord wrapping on intracardiac blood circulation in the fetus. Our previous study [22] showed no significant differences for Tei RV values (0.5 ± 0.2 vs. 0.4 ± 0.1) as well as for the components of the Tei index, i.e., isovolumetric contraction time, isovolumetric relaxation time, and ejection time. Isovolumetric contraction time is the period during which the ventricles contract but the volume of the chamber remains constant due to the fact that all the valves are closed. Isovolumetric relaxation time is the period during which the ventricles relax but the volume of the chamber again remains constant due to the fact that all the valves are closed. Ejection time is the duration of systole during which blood is pumped out of the heart into the aorta [23].

The values were as follows: Tei ET RV: 166.2 ± 21.3 vs. 169.1 ± 19.7; Tei LV: 0.5 ± 0.1 vs. 0.5 ± 0.1; Tei ET LV: [ms] 161.3 ± 19.3 vs. 163.1 ± 15.60; Tei ICT LV: [ms] 41.4 ± 15.4 vs. 38.9 ± 12.5; Tei IRT LV: [ms] 43.5 ± 12.4 vs. 42.3 ± 9.6.

All measurements were performed at 28 weeks and 5 days of pregnancy in 38 fetuses wrapped once with the umbilical cord around the neck compared to 77 fetuses from the control group [22]. The same results were confirmed for 46 fetuses wrapped with one coil of the umbilical cord around the neck compared with 70 fetuses without being wrapped with the umbilical cord at 28.1 weeks of pregnancy. The values were as follows: Tei RV: 0.51 ± 0.2 vs. 0.48 ± 0.2; Tei LV: 0.54 ± 0.15 vs. 0.5 ± 0.12.

There were also no significant differences between the study groups [19]. Based on the study at 31.3 weeks of pregnancy, the Tei index value seemed not to be of any importance. There was no significance between the Tei index value for 139 fetuses with a single nuchal cord and 158 fetuses from the control group. The values were as follows: Tei RV: 0.5 ± 0.2 vs. 0.5 ± 0.2 and Tei LV: 0.5 ± 0.1 vs. 0.5 ± 0.2 [21].

Only significantly higher Tei RV values were obtained in fetuses with LGA: (25 cases) 0.6 ± 0.2 vs. 0.5 ± 0.2 (*p* = 0.01) and in the case of Tei LV values in a group of 11 cases of LGA fetuses wrapped around the neck compared to LGA fetuses without umbilical cord around the neck: 0.5 ± 0.1 vs. 0.6 ± 1 (*p* = 0.009) [21]. These results were so ambiguous that it was difficult to assess whether fetuses with LGA wrapped with the umbilical cord really had lower ventricular systolic and diastolic parameters. A larger group of LGA fetuses wrapped with the umbilical cord around the neck would be needed. However, the results of this study indicate significantly higher Tei index values for the left and right ventricles of the heart not only in fetuses with two coils of the umbilical cord around the fetal neck, but also in fetuses with one coil (Figure 1, Table 1). It results from a significant increase in the number of fetuses in the study group (680 fetuses without umbilical cord, 93 wrapped once, and 26 wrapped twice) and performance of examination in a later period of pregnancy, i.e., at 33.1 weeks of pregnancy. It is believed that if the fetus is wrapped with the umbilical cord around the fetal neck, the vagus nerve can potentially be affected. The vagus nerve is a cranial nerve that goes from the brain to the abdomen and passes through the neck. If the umbilical cord is found around the fetal neck, it may exert pressure on the vagus nerve, which potentially impacts the nerve function and affects the fetal heart function [24,25,26], which is indicated by an increased Tei index.

The mechanisms by which nuchal cords can affect cardiac function may also include the potential compression of the carotid arteries caused by the umbilical cord wrapping. This compression can lead to an increase in blood pressure, which may result in the redistribution of blood flow. The observed changes, such as fetal heart enlargement and disproportion, widening of the pulmonary artery, right ventricle, right atrium, and myocardial hypertrophy, could be related to this redistribution of blood flow due to the compression of the carotid arteries. In the context of our previous study presented in Diagnostics 2023 [18], based on the findings of the study and the proposed mechanisms of how the nuchal cords affect cardiac function, it is likely that the observed changes are a direct result of the nuchal cord presence rather than just a correlation. The findings suggest that the entanglement of the umbilical cord around the neck can affect the vagus nerve, leading to alterations in the Tei index values, which reflect the systolic and diastolic function of the heart chambers. The increased Tei index rates observed in fetuses with one or two coils of the umbilical cord around the neck, compared to those without cord entanglement, support the direct impact of the nuchal cord on fetal cardiac function. Further research and studies are needed to explore the specific mechanisms by which nuchal cords affect cardiac function and to determine the causative relationship between umbilical cord entanglement and changes in fetal heart circulation.

Wrapping of the fetal neck with the umbilical cord, known as a nuchal cord, is commonly associated with fetal movements. Fetal movements can result in cord entanglement, with excessive fetal movements and long umbilical cords being prone to entanglement. However, it is not entirely clear why some fetuses develop nuchal cords while others do not. Factors such as fetal position, the length of the umbilical cord, and fetal activity levels may play an important role in the formation of nuchal cords. Increased fetal surveillance may be indicated in certain clinical conditions that are further complicated by decreased fetal movements, decreased amniotic fluid volume, postdates, fetal growth restriction, and impaired umbilical artery Doppler flow velocimetry [27,28,29]. The insights gained from this study and our previous study (Diagnostics 2023) [18] regarding the effect of umbilical cord wrapping around the fetal neck on fetal circulation and cardiac remodeling could influence monitoring and management strategies during pregnancy and labor in the following ways:

1. Increased Monitoring: Pregnant women with fetuses showing multiple nuchal cords may require more extensive monitoring of fetal circulation and cardiac parameters, such as echocardiographic assessments. This could help in the early detection of any abnormalities and timely intervention if required.
2. Specialized Monitoring: Specialized monitoring techniques, such as assessing the Tei indexes, Pa/Ao ratio, heart area/chest area ratio, and global sphericity index, could be incorporated into routine prenatal care for women with fetuses wrapped with the umbilical cord. This could provide a more comprehensive evaluation of fetal well-being.
3. Polyhydramnios Evaluation: Our previous studies [18] suggest a correlation between umbilical cord wrapping and polyhydramnios. Obstetricians may consider monitoring amniotic fluid levels more closely in such cases to ensure optimal management.
4. Fetal Growth Assessment: The study [18] highlights the importance of evaluating fetal growth and circulation in cases of umbilical cord wrapping. Monitoring fetal growth and vascular parameters could help in assessing the impact of cord compression on fetal well-being and growth.
5. Antenatal Counseling: Obstetricians could use the insights from our studies [18,19,21,22] to provide more targeted and specific counseling to pregnant women with fetuses wrapped with the umbilical cord. This could include discussing the potential implications of such wrapping on fetal circulation and cardiac development.

The strengths of this article include the following:

- Innovative Approach: this study introduces a novel perspective on functional echocardiographic analysis of the fetal heart, particularly in cases where the umbilical cord is wrapped around the fetal neck.
- Vagus Nerve Stimulation Hypothesis: it presents a compelling hypothesis on how the umbilical cord wrapping could stimulate the vagus nerve, offering a fresh insight into fetal development.
- Prenatal Focus: by concentrating on prenatal analysis, this study fills a gap in the literature, which often focuses on postnatal outcomes following umbilical collision scenarios.
- Reader Engagement: the unique subject matter concerning prenatal echocardiographic analysis is likely to captivate readers and stimulate new discussions in the field.
- Potential for Clinical Impact: the insights provided could have significant implications for prenatal care and the management of umbilical cord complications during pregnancy.

In spite of having a large database of fetuses, some weaknesses of our study include the following:

- Limited Scope: the research is based on a small sample size, which may not provide a comprehensive view of the subject matter.
- Need for Multi-Center Collaboration: engaging multiple research centers could enhance the validity and reliability of the study through varied data collection and analysis.
- Prospective Study Design: future research could benefit from a prospective approach, allowing for real-time data gathering and potentially more accurate results.
- Clinical Practice: the current study’s findings may have limited immediate applicability in clinical settings due to the aforementioned constraints.

## 5. Conclusions

Umbilical cord wrapping around the neck may influence fetal heart study without any mechanically induced compression of the umbilical vessels in normal pregnancies. These findings suggest a mechanism of protection acting against the restriction of the umbilical vessel area and interference with the fetal blood circulation. Further research is needed to substantiate that the wrapping of the cord causes the changes in cardiac function, not just correlation. Given these findings, it is imperative that future research endeavors focus on elucidating the precise mechanisms by which nuchal cords may affect cardiac function. This includes investigating whether the changes observed are a direct result of the cords’ presence or merely a correlation. Such studies should aim to determine the clinical significance of these findings and whether they warrant a change in monitoring and management strategies during pregnancy and labor. While nuchal cords are common and not typically associated with serious complications, their potential impact on fetal heart function is an important subject for further research. This research should seek to clarify the relationship between nuchal cords and cardiac function, to improve our understanding and management of these cases in clinical practice.

## Figures and Tables

**Table 1:** Median values of Tei index for the left and right ventricles in reference to the number of coils of the umbilical cord around the fetal neck and statistical significance (statistically significant difference).

33w1d Mean GA	Fetuses with 0 Nuchal Cords—680 Cases	Fetuses with 1 Nuchal Cord—93 Cases	*p*-Stat. Significant Difference	Fetuses with 0 Nuchal Cords—680 Cases	Fetuses with 2 Nuchal Cords—26 Cases	*p*- Stat. Significant Difference
Tei RV (median)	0.40	0.44	0.000001	0.40	0.55	0.000001
Tei LV (median)	0.40	0.43	0.00025	0.40	0.50	0.0015

**Table 2:** Median values of PI UMBA in reference to the number of coils of the umbilical cord around the fetal neck (no statistical significance).

33w1d Mean GA	Fetuses with 0 Nuchal Cords—680 Cases	Fetuses with 1 Nuchal Cord—93 Cases	Fetuses with 2 Nuchal Cords—26 Cases	*p*- Stat. Significant Difference
PI UMBA (median)	1.00	0.95	0.92	0.3179

## Data Availability

The original contributions presented in the study are included in the article/Supplementary Materials, further inquiries can be directed to the corresponding authors.

## Supplementary Materials

The following supporting information can be downloaded at: https://www.mdpi.com/article/10.3390/jcm13226836/s1. Supplementary Clip S1: Single nuchal cord formed in U shape—fetal cross-section, Doppler color. Supplementary Clip S2: Double nuchal cord formed in U shape—fetal cross-section, Slow flow HD. Supplementary Clip S3: Double nuchal cord formed in U shape—fetal longitudinal section, Doppler color.
